# Working-Together Normative Appeals to Promote Pro-Environmental Donations

**DOI:** 10.3390/bs14040273

**Published:** 2024-03-26

**Authors:** Yanlin Wu, Yue Chen, Cancan Jin, Junsheng Qin, Lei Zheng, Yidi Chen

**Affiliations:** 1 Department of Psychology, School of Humanities and Social Sciences, Beijing Forestry University, Beijing 100083, China; wuyanlin2023@m.scnu.edu.cn (Y.W.); chenyue@bjfu.edu.cn (Y.C.); jincancan@bjfu.edu.cn (C.J.); qinjunsheng@bjfu.edu.cn (J.Q.); 2 School of Business, Macau University of Science and Technology, Macau 999078; leizh@pku.edu.cn

**Keywords:** working-together normative appeals, pro-environmental donations, intentions towards pro-environmental donations, mediating effect

## Abstract

Investigating the role of social norms in fostering pro-environmental behaviors is crucial for advancing human efforts toward environmental protection. This study employed a one-way, two-level, between-participants experimental design, focusing on the type of social norm as the independent variable and pro-environmental donations as the dependent variable. This study aimed to explore the impact of working-together normative appeals on pro-environmental donations and to understand the underlying mediating mechanism. In total, 128 Chinese university students participated in an online experiment. The findings indicated that working-together normative appeals significantly increased both the intention to donate and the actual amount of pro-environmental donations in the experimental group compared to those in the control group. Furthermore, the perceived behavioral control and intentions towards pro-environmental donations were identified as mediators in the relationship between social norm categorization and pro-environmental donations. Notably, intentions towards pro-environmental donations alone had a substantial mediating effect. These results underscore the positive influence of working-together normative appeals on pro-environmental donations and offer valuable insights into encouraging active participation in the creation of an eco-friendly society, particularly within a collectivist cultural context.

## 1. Introduction

Ecological and environmental problems have attracted considerable attention worldwide and have become some of the most critical issues of our time [1]. Unsustainable global practices have led to a host of critical environmental issues, encompassing climate change, escalating global warming, pervasive pollution, the depletion of the ozone layer, significant loss of biodiversity, widespread deforestation, and increasing desertification. These challenges underscore the urgent need for a collective shift toward more sustainable and environmentally conscious behaviors on a global scale [2]. As indicated by the 2022 Environmental Performance Index, China holds the 160th position out of 180 countries. This ranking highlights the deficiencies in China’s environmental protection strategies and its efforts toward sustainable remediation. Additionally, this positioning underscores the need for more robust and effective measures to enhance China’s environmental performance on a global scale [3]. Therefore, the prevention of environmental risk factors is particularly important [2]. Preventing environmental risk factors is particularly challenging, but a sustainable ecological environment is a prerequisite for human development [4].

Numerous challenges, including the depletion of natural resources, ecological degradation, and widespread environmental pollution, pose considerable threats to the sustainable development of humanity [1], and many researchers have emphasized the importance of promoting individual pro-environmental behaviors [5]. Therefore, defining and assessing pro-environmental behaviors is of particular value. Because individuals have an instinct to secure their material benefits, measuring an individual’s pro-environmental level in terms of donation behaviors can go a long way toward capturing their pro-environmental orientation compared with other forms of measurement [6].

China’s population size and global economic status make its environmental footprint substantial, highlighting the importance of addressing this issue. College students are at a critical period for the development of environmental awareness and behaviors, and gain financial autonomy as they enter university. Therefore, this study aimed to explore the influence of social norms on pro-environmental donations among college students. Our work contributes to the literature in three ways. First, we demonstrate the effectiveness of the “Working-together” normative appeals for pro-environmental donations in a collectivist culture. Second, the current study was conducted to enrich the social identity model of pro-environmental action (SIMPEA) and the mind sponge theory [7,8]. Third, this study supplemented the content of the theory of planned behavior [9].

## 2. Literature Review and Hypotheses

### 2.1. Pro-Environmental Behaviors and Pro-Environmental Donations

There are various definitions of pro-environmental behaviors in scholarship, but they all point to the same connotation of “increasing behavior that is beneficial to the environment and minimizing behavior that is harmful to the environment”, which is in contrast to anti-pro-environmental behaviors [10]. Hunter et al. classified pro-environmental behaviors into “private” pro-environmental behaviors (e.g., garbage classification, and water conservation) and “public” pro-environmental behaviors (e.g., participation in environmental protection organizations, and donations to environmental protection activities) [11]. Environmental protection is related to the welfare of individuals and society, and it is necessary to achieve effective social promotion of pro-environmental behaviors [12,13]. The concept of pro-environmental behavior overlaps with the concept of pro-social behavior in some areas. Pro-social behaviors represent a broad category of acts that are defined by significant regions of society as generally beneficial to other people or one’s group [14]. Pro-social behavior involves trade-offs between our own well-being and the well-being of others. Pro-social behaviors that involve a commitment to the environmental sphere are also classified as pro-environmental, such as donations to environmental organizations.

The self-report method is the preferred method for measuring pro-environmental behaviors, having been adopted by most researchers [15]. However, self-reported pro-environmental behaviors often do not accurately represent the actual pro-environmental behaviors of individuals. Research has shown low consistency between self-reported pro-environmental attitudes or behaviors and actual objective behaviors [16]. To avoid the measurement bias of self-reporting, many studies have used experiments to measure the real environmental behaviors of participants by reproducing scenes from their daily lives. Pro-environmental donations measured by voluntary donations of money are widely used in experimental studies and are a common means of measuring altruistic behaviors because this measure can approach the reality of charitable giving [17]. According to self-perception theory [18] and the moral credentials hypothesis of moral licensing effects [19], when people are aware of the environmental significance and moral value of environmentally friendly charitable giving behaviors, they can experience positive emotions that contribute to the longevity of environmentally responsible practices [20].

### 2.2. Social Norms and Pro-Environmental Behavior

Most pro-environmental behaviors take place at a collective level [21,22,23]. Fritsche et al. [7] realized that the existing pro-environmental research focuses too much on individual factors and lacks a mechanism to explore pro-environmental behaviors from a social identity perspective. They therefore argued that there is a need to expand the theoretical framework of exploring the causes and mechanisms of pro-environmental behaviors beyond the individual cognitive perspective to reveal the causes and mechanisms of pro-environmental behaviors from a broader group cognitive perspective. In fact, people are more likely to be influenced by those they perceive as belonging to their own group. For example, when a scientist who favors the use of recycled water emphasizes their shared regional perceptions rather than de-emphasizing their own regional identity, those who identify with a particular region are more likely to support the use of recycled water [24]. This suggests that environmental attitudes and behaviors are more likely to be changed by those who consider themselves as members of a group. Another study suggests that leaders who advocate for renewable energy and emphasize shared group membership (e.g., use of “we”) are more likely to influence people’s intentions to use renewable energy than those who do not use such statements [25]. This would not only extend the theoretical capacity to explain people’s responses to global environmental events but also contribute to the realization of effective environmental governance.

Social norms can be categorized into several types. Based on previous research syntheses, in terms of the components that constitute social norms, they can be distinguished into descriptive norms and injunctive norms. Descriptive norms refer to the behavior of the majority, while injunctive norms refer to behaviors that are approved by the majority. Based on the validation of social norms, they can be divided into actual norms and perceived norms. Furthermore, based on the emphasis of social norms on appropriate ideal behavior, social norms can be divided into prescriptive norms and proscriptive norms. In the literature concerning social norms and pro-environmental behavior, the most researched distinction seems to be between injunctive norms and descriptive norms [26]. Working-together normative appeals are a social norm presented from a social perspective that includes descriptive norms about how most people do things and invites others to join. Howe et al. found working-together normative appeals to be the most effective way to encourage behavioral change [27]. Working-together normative appeals involve the inclusion of a “doing-it-together” component in the presentation of social norms, effectively enhancing individuals’ willingness to engage in charity and boosting the actual amount of donations by inviting them to “join in”. The social identity model of pro-environmental action (SIMPEA) highlights the significant influence of group dynamics in fostering pro-environmental behaviors [7]. This model emphasizes the interaction of in-group identification, the norms and objectives of the group, and collective efficacy, all of which are activated at the group level, as pivotal in shaping pro-environmental actions. When an individual psychologically identifies themselves with a certain group, they perceive and adhere to the ingroup social norms [28]. When individuals highly recognize their affiliation with a group, the group norms increase the likelihood of in-group members acting in accordance with those social norms [29].

The mind sponge theory posits that individuals, upon receiving external information, judge whether this information falls within their comfort zone based on their own values and core beliefs. Then, they filter and process the information to make decisions that are more aligned with their values [8]. Chinese culture emphasizes collectivism and places greater importance on the long-term collective social benefits [30]. Therefore, when individuals receive invitations from groups with identities similar to their own, it activates the internal collectivist values of the Chinese people, leading them to make decisions consistent with their group. Working-together normative appeals cooperatively build on the context of collective action to change the perception of factors that promote pro-environmental donations by creating a sense of cooperation toward a common goal, potentially activating individuals’ internal motivations. Additionally, beyond the influence of group social norms, working-together normative appeals can also reduce the perceived command tone, thereby alleviating the social pressure associated with the appeal and promoting the formation of social norms. Previous research suggests that this approach appears to invite you to join others in a collective effort for a common good [31]. Furthermore, reviews in the field of health behavior indicate that compared to a gentler expression, commands often yield less favorable outcomes [32].

**Hypothesis** **1:** *The type of social norm invoked will differ in pro-environmental donations and working-together normative appeals will produce higher levels of pro-environmental donations than social norms that do not include working-together normative appeals.*

### 2.3. Mediating Role of Perceived Behavioral Control and Pro-Environmental Intentions

Howe et al. have also pointed out that intermediate mechanisms are involved in the process, whereby working-together normative appeals affect pro-environmental donations, and these mediating mechanisms need to be explored in more detail [27]. In environmental research, the theory of planned behavior posits that social norms influence pro-environmental intentions through three key components: behavioral attitudes, subjective norms, and perceived behavioral control, which, in turn, drive pro-environmental behaviors [9]. This suggests that social norms are a fundamental factor in shaping environmental actions. Perceived behavioral control, as defined by [33], refers to an individual’s assessment of the ease or difficulty of performing a specific behavior, reflecting their perception of enabling or hindering factors. Numerous studies have employed this theory to investigate the mechanisms and influencers of pro-environmental behaviors [34,35,36]. A general consensus emerged that perceived that behavioral control positively predicted pro-environmental behaviors [37,38,39]. A previous study found that perceived behavioral control mediated the relationship between social norm and pro-environmental behaviors [40]. Furthermore, perceived behavioral control has been identified as a crucial direct determinant of pro-environmental intentions [41,42]. Given this context, the role of social norms in pro-environmental donations appears to be quite complex, necessitating the introduction of multiple mediation models (such as the serial mediation model) to explain their relationship [43]. Considering this backdrop, we have introduced the concept of chain mediation to explain the relationship between working-together appeals and pro-environmental donations. The chain mediation model refers to a situation where there is an impact relationship between mediating variables, with the mediating variables exhibiting sequential characteristics, forming a mediation chain [44]. Therefore, the current study aimed to explore the chain mediating effect of perceived behavioral control and intentions towards pro-environmental donations in the impact of the social norm type on pro-environmental donations.

**Hypothesis** **2:** *Social norms could impact intentions towards pro-environmental donations and, consequently, pro-environmental donations through the lens of perceived behavioral control.*

## 3. Methods

### 3.1. Participants

For the planning of the sample size in this study, G*Power 3.1 software was utilized. Setting the effect size (d) at 0.50, the alpha level at 0.05, and the power at 0.80, the software estimated that a total of 128 participants would be required for the experiment, with an average of 64 participants per group. Recruitment was conducted online, with recruitment advertisements distributed via two online platforms for recruiting participants for psychological experiments (Passion Experiment Participant Recruitment and Online Participant Recruitment) and WeChat Moments. The inclusion criteria for recruitment were as follows: (1) undergraduate students in China, (2) proficient in using smartphones, and (3) at least 18 years of age. A total of 148 participants were recruited. Of these, five were excluded because they failed the attention test questions during the completion of the scale, two were excluded because they reported that they were completely or relatively unable to understand the meanings expressed by the leaflets during the experiment, and thirteen were excluded because they were unsuccessfully manipulated. The attention test consisted of one question: “Please calculate the result of 6 minus 4”. The final number of valid participants was thus 128 (53 female), with a mean age of 20.75 years (*SD* = 1.25 years), with 70 in the experimental group and 58 in the control group. This study was approved by the Ethics Committee of Beijing Forestry University.

### 3.2. Experimental Design

A one-way two-level (working-together normative appeals group vs. social norms control group) between-participants experimental design was used, with the type of social norm as the independent variable and intentions towards pro-environmental donations (intentions to donate) and pro-environmental donations (amount of donation) as the dependent variables.

### 3.3. Materials

#### 3.3.1. Manipulation of Independent Variables

**Figure 1:**
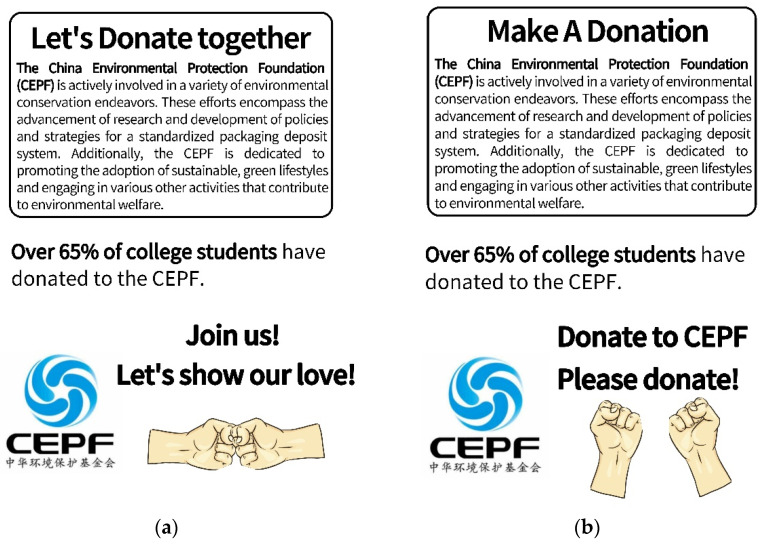
Posters of working-together normative appeals and normative appeals (**a**), working-together normative appeals group, (**left**); (**b**), control group, (**right**).

The paradigm used was based on Howe et al., in which the working-together normative appeals group was manipulated using a poster titled “Let’s Work Together”, which included the elements “Join Us” and “Donate Together” and a picture of “Fist bumps” (Figure 1a) [27]. The control group was manipulated using a poster titled “Compassion Donates” containing only a social norm message and a picture of “two separate hands” (Figure 1b). The manipulation check was carried out via one item “If you were to donate to this foundation, to what extent do you feel you would be donating alongside other university students?”. Participants who failed to perceive themselves donating together with others in the working-together condition, and in the control condition, those that felt that they were donating together with others were excluded from the final analysis.

#### 3.3.2. Perceived Behavioral Control

Drawing on Barbera et al.’s work, perceived behavioral control was measured using two items: “I have the ability to participate in garbage collection on a regular basis” and “For me, participating in garbage collection on a regular basis is doable”. Scoring was based on a 5-point scale, with 1 representing strongly disagree and 5 representing strongly agree [45]. Higher scores represented greater perceived behavioral control.

#### 3.3.3. Pro-Environmental Donations and Intentions towards Pro-Environmental Donations

Referring to the pro-environmental behavior game task paradigm [46], intentions towards pro-environmental donations were measured by the question, “If you see this flyer, do you want to donate to this recycling organization?” To measure the participants’ intention to donate, a 7-point scale was used, ranging from “very unwilling” to “very willing”. Pro-environmental donations were rated on a 6-point scale, in which participants were informed in advance that the reward for the experiment was RMB 5, and were asked how much of that fee (RMB 0–5) they would be willing to donate to the China Environmental Protection Foundation. The participants were advised that if they were willing to make a donation, the corresponding donation amount would be deducted from the participant fee.

#### 3.3.4. Demographic Information

Demographic information collected from the participants included gender and age.

### 3.4. Procedures

**Figure 2:**
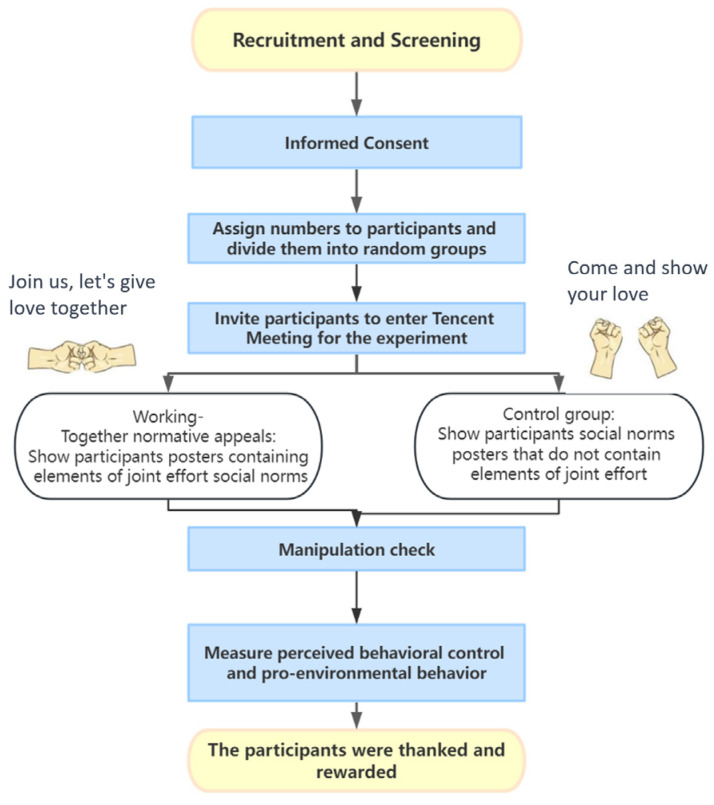
The procedure of this research.

The participants were recruited to participate in experiments by signing an informed consent form. The experimental procedure was based on the study conducted by Howe et al., in which prior to the start of the experiment, each subject was numbered and randomly assigned to a control group (social norms) or an experimental group (working-together normative appeals) [27]. This was followed by the start of the formal experiment in an online meeting room, a manipulation check at the end of the experiment, and the completion of the measurement of the remaining variables. The participants were paid RMB 5 upon the completion of the experiment; the exact experimental procedure is shown in Figure 2.

### 3.5. Data Analysis

SPSS (version 26.0) was used for descriptive statistics and correlation analysis, independent samples *t*-test, and mediation effect analysis. Mediation models were established using Macro Model 6 of the PROCESS macro of SPSS, and 5000 bootstrap tests for mediation effects were conducted. Visualization was performed using the ggplot2 and ggsignif packages for R.

## 4. Results

### 4.1. Manipulation Check

A manipulation check was performed using the question “If you donate to this foundation, do you feel that you are donating with other college students” (1 for complete disagreement, 7 for complete agreement). An independent samples *t*-test showed that the scores of the experimental group (*M* = 4.26, *SD* = 0.70) were significantly higher than those of the control group (*M* = 3.45, *SD* = 0.92), *t*(126) = 5.51, *p* < 0.001, suggesting that the experimental manipulation was successful.

### 4.2. The Effect of Social Norm Type on Perceived Behavioral Control

**Figure 3:**
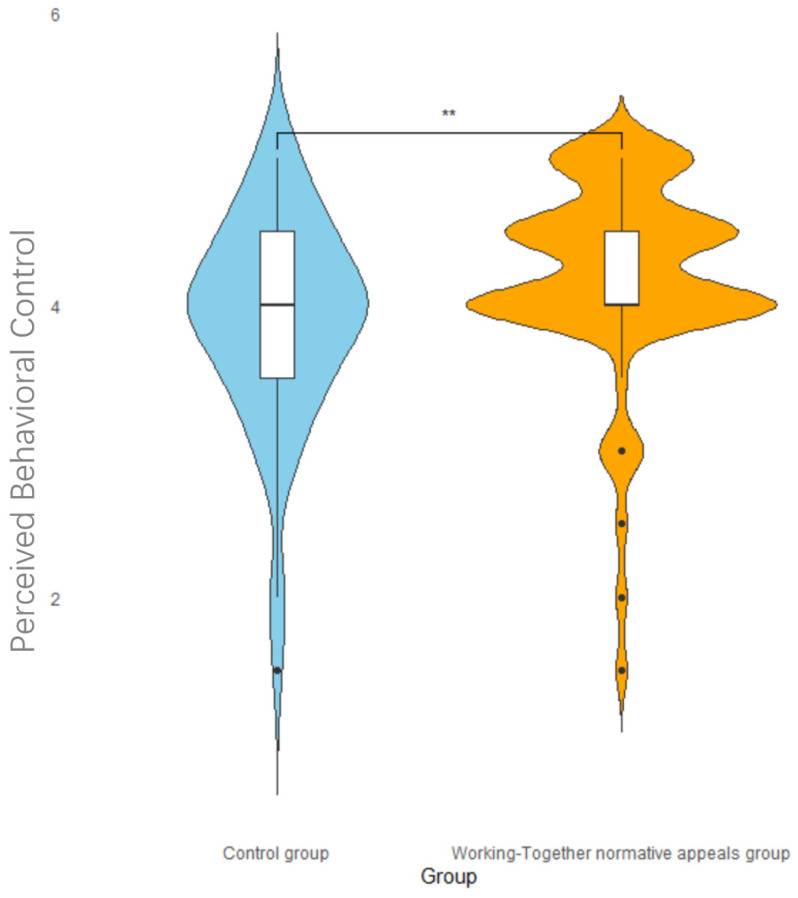
The impact of social norm grouping on perceived behavioral control. Note: ** *p* < 0.01.

An independent sample *t*-test with the social norm type as the independent variable and perceived behavioral control as the dependent variable showed a significant difference in social norm types, *t*(126) = −2.32, *p* = 0.022, Cohen’s d = 0.59. The experimental group scores (*M* = 4.19, *SD* = 0.69) were significantly higher than the control group scores (*M* = 3.90, *SD* = 0.72), suggesting that the social norm type of manipulation can significantly enhance the level of an individual’s perceived behavioral control. The distribution of perceived behavioral control among the two groups is shown in the violin plot below (Figure 3). It can be concluded that working-together normative appeals can enhance perceived behavioral control. The scores for most of the participants in the experimental group were distributed above four points, while the participants in the control group were distributed above and below four points.

### 4.3. The Effect of Social Norm Type on Intentions towards Pro-Environmental Donations

**Figure 4:**
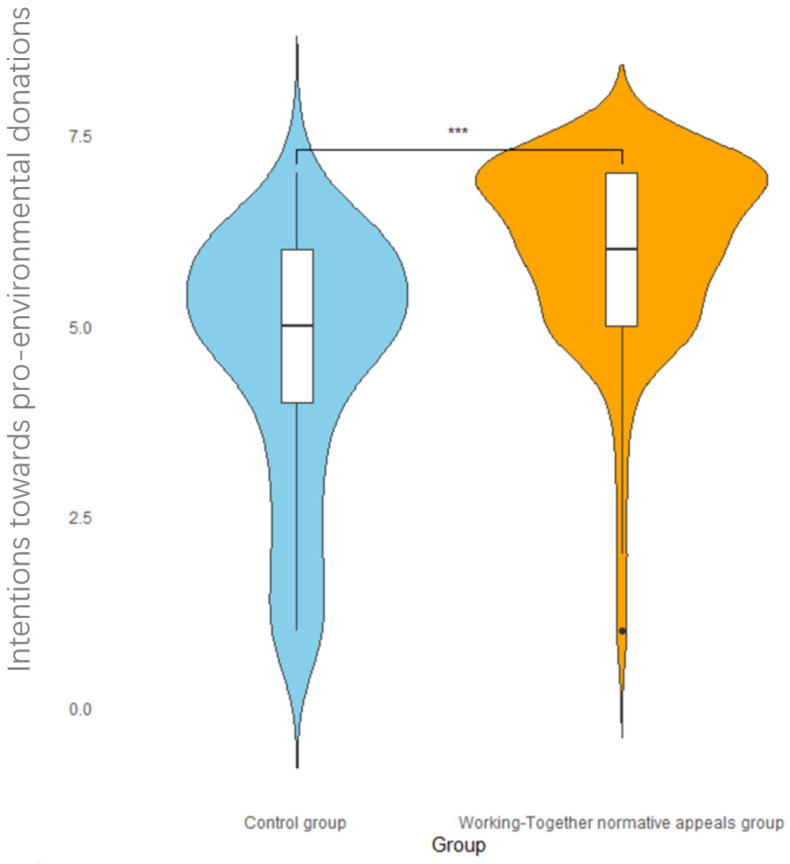
The impact of social norm grouping on intentions towards pro-environmental donations. Note: *** *p* < 0.001.

An independent sample *t*-test with social norm type as the independent variable and intentions towards pro-environmental donations as the dependent variable found that the intentions towards pro-environmental donations of the experimental and control groups were significantly different, *t*(104) = 5.41, *p* < 0.001, Cohen’s d = 0.97. The intentions towards pro-environmental donations of the experimental group (*M* = 6.00, *SD* = 1.23) were significantly higher than those of the control group (*M* = 4.59, *SD* = 1.64). The violin plot of the distribution of intentions towards pro-environmental donations is shown in Figure 4, in which it can be seen that working-together normative appeals can enhance the participants’ intentions to donate to environmental protection organizations; most of the scores for participants in the experimental group were distributed above five points, while the control group’s participants’ scores were distributed above and below five points.

### 4.4. The Effect of Social Norm Type on Pro-Environmental Donations

**Figure 5:**
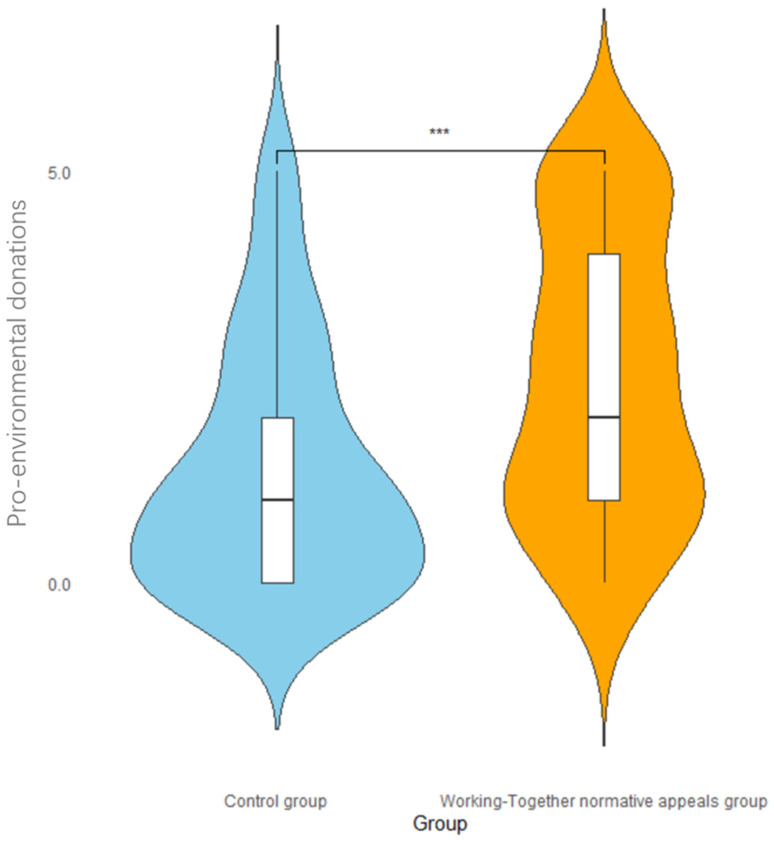
The impact of social norm grouping on pro-environmental donations. Note: *** *p* < 0.001.

An independent sample *t*-test with social norm type as the independent variable and pro-environmental donations (donation amount) as the dependent variable revealed a significant difference between the donation amount of the experimental and control groups, *t*(126) = 3.95, *p* < 0.001, Cohen’s d = 0.70. The score of the experimental group (*M* = 2.47, *SD* = 1.71) was significantly higher than that of the control group (*M* = 1.36, *SD* = 1.47). The violin plot of the distribution of pro-environmental donations is shown in Figure 5, in which it can be seen that the social norm of working-together can enhance the participants’ donation amounts to environmental organizations, with most of the participants’ donations in the experimental group being roughly evenly distributed in the range of RMB 1–5, while the participants’ donations in the control group were mostly distributed in the range of RMB 0–1.

### 4.5. Chain Mediation Model

Using the type of social norm as the independent variable (working-together norms = 1, no working-together normative–information social norms = 0), perceived behavioral control and intentions towards pro-environmental donations as mediating variables, pro-environmental donations as the dependent variable, and gender and age as covariates, 5000 samples were extracted using the bootstrapping method to for 95% confidence interval (95% CI) estimations. To reduce multicollinearity [47], in reference to previous studies [48,49], all data have been mean-centralized, and the following results are presented as standardized coefficients. The predictive effect of the type of social norms on perceived behavioral control was significant, β = 0.20, SE = 0.09, *p* = 0.022, 95% CI = [0.03, 0.38]. Furthermore, the predictive effect of the type of social norms on intentions towards pro-environmental donations was significant, β = 0.39, SE = 0.08, *p* < 0.001, 95% CI = [0.24, 0.55]. The predictive effect of perceived behavioral control on intentions towards pro-environmental donations was significant as well, β = 0.25, SE = 0.08, *p* = 0.002, 95% CI = [0.10, 0.41]. The predictive effect of the type of social norms on pro-environmental donations was not significant, β = 0.16, SE = 0.09, *p* = 0.072, 95% CI = [−0.01, 0.34]. The predictive effect of perceived behavioral control on pro-environmental donations was not significant, β = 0.13, SE = 0.08, *p* = 0.111, 95% CI = [−0.03, 0.30]. Furthermore, the predictive effect of intentions towards pro-environmental donations on pro-environmental donations was significant, β = 0.32, SE = 0.09, *p* < 0.001, 95% CI = [0.13, 0.50].

**Figure 6:**
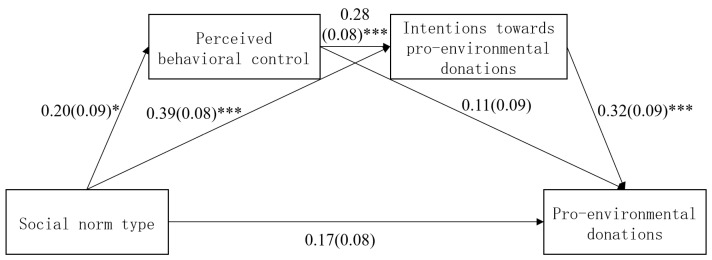
Chain mediation effect model. Note: * *p* < 0.05, *** *p* < 0.001.

The results showed that the total indirect effect was significant, as the standardized effect value was 0.17, SE = 0.05, 95% CI = [0.08, 0.27]; indirect path 1 (simple mediation: type of social norms → perceived behavioral control → pro-environmental donations) was not significant, as the standardized effect value was 0.02, SE = 0.02, 95% CI = [−0.005, 0.07]; indirect path 2 (chain mediation: social norm type → perceived behavioral control → intentions towards pro-environmental donations → pro-environmental donations) was significant, as the standardized effect value was 0.02, SE = 0.01, 95%CI = [0.004, 0.05]; and indirect path 3 (simple mediation: social norm type → intentions towards pro-environmental donations → pro-environmental donations) was significant, as the standardized effect value was 0.12, SE = 0.04, 95% CI = [0.05, 0.22]. The direct effect of social norm type on pro-environmental donations was not significant, as the standardized effect value was 0.17, SE = 0.09, 95% CI = [−0.003, 0.34]. The mediation model is shown in Table 1 and Figure 6.

The above mediation effect analysis indicates that participants subjected to joint-effort information initiation exhibited higher perceived behavioral control, enhancing pro-environmental intention and pro-environmental behaviors, compared to the control group, which received only social norm information. Joint-effort information initiation was also found to enhance pro-environmental behaviors directly through pro-environmental intention.

## 5. Discussion

This study successfully developed an ecologically valid instrument to determine the effectiveness of working-together normative appeals in initiating more intentions and actual behaviors towards pro-environmental donations in participants, validating H1. Additionally, this study showed that working-together normative appeals can strengthen perceived behavioral control, which, in turn, fosters pro-environmental donations through the intentions towards pro-environmental donations, partially validating H2. With regard to the SIMPEA, the current study adapted the research paradigm of Howe et al. by combining working-together normative appeals with a donation paradigm for measuring pro-environmental behaviors to examine the effect of working-together normative appeals on pro-environmental donations in a more ecologically valid everyday real-world context [27]. In terms of measurement, to ameliorate the problem, i.e., how previous studies tended to rely on self-reporting in the measurement of pro-environmental behaviors, which can reduce the ecological validity of the measurements in this field [15], the present study used a real decision-making situation and a donation paradigm to gauge pro-environmental behaviors in actual settings.

### 5.1. Working-Together Normative Appeals to Promote Pro-Environmental Donations

The results showed that participants in the working-together normative appeals group chose to donate more to the China Environmental Protection Foundation than those in the social norms control group. This is consistent with previous findings [27], suggesting that conveying social norms that invite people to work together for environmental protection is more likely to stimulate people’s intentions to donate than social norms that only request that people donate for environmental protection.

This result can be explained by the SIMPEA, which suggests that people act in accordance with the norms of a particular group when they develop an emotional attachment and psychological identification with that group [7]. That is, group norms are formed on the basis of an individual’s positive psychological identification with the group to which they belong [28], and group norms cause individuals to acquire in-group perceptions, behavioral norms, and goals [50,51], leading to behavioral changes. The use in posters of messages such as “Let’s work together”, “Join us”, and “Donate together”, which represent invitation, cooperation, and joint efforts, encourages individuals to develop a positive sense of identification with the group to which they belong. These messages also lead individuals to realize that both the group they belong to and they themselves are making efforts to donate to the environment, thus unintentionally stimulating the initiative of individuals to socially interact with the group to which they belong and promoting their pro-environmental behavior [27].

Social information processing theory can be used to explain the success of posters that incorporate information about working-together normative appeals. The theory emphasizes the role of environmental factors in the development of individual behavior, in which people process and interpret specific social information to form cognitions and behaviors, adapting attitudes, behaviors, and beliefs to appropriate environmental cues [52,53,54]. Working-together normative appeals encourage them to construct an environmental image of the group, which promotes a pro-environmental climate within the group and, in turn, pro-environmental behaviors in individuals [52,55]. Person–context interaction theory suggests that an individual’s behavior develops and is shaped by the interaction between the individual and the environment [56]. The results of this study show that the factors influencing an individual’s pro-environmental behavior include not only psychological factors (e.g., attitudes) and external factors (e.g., social norms), but also the results of the individual’s psychological interactions with social groups.

### 5.2. Explanatory Mechanisms for Perceived Behavioral Control and Intentions towards Pro-Environmental Donations

This study revealed that perceived behavioral control and intentions towards pro-environmental donations serve a sequential mediating role between types of social norms and pro-environmental donations, thereby corroborating the theory of planned behavior. This theory posits that behavioral intention is the most direct determinant of behavior, which is influenced by perceived behavioral control. Additionally, it asserts that control beliefs, encompassing social norms, are the primary antecedents of behavior. Successful behavior execution hinges on both favorable intentions and an adequate level of perceived behavioral control. Furthermore, this study observed a significant standalone mediating effect of intentions towards pro-environmental donations, aligning with previous findings [27]. This indicates that the combined impact of social norms as influential control beliefs can foster pro-environmental donations by shaping intentions.

Contrary to our hypothesis, the direct effect of working-together normative appeals on the prediction of pro-environmental donations was not significant after including perceived behavioral control and intentions towards pro-environmental donations in the model. Baron and Kenny argued that the presence of complete mediation represents the most robust evidence of a mediating effect [57] and negates the need for future exploration of alternative mediators [58], demonstrating the validity of the explanatory power of chain mediation.

In addition, according to the theory of planned behavior, perceived behavioral control can replace actual behavioral control to a certain extent and can be used to predict people’s behavior. However, this study did not find a separate mediating effect of perceived behavioral control, which may be related to the fact that, under the SIMPEA, other variables may interact with working-together normative appeals [12,13]. The formation of pro-environmental behaviors is the result of multiple psychological variables working together, and according to the SIMPEA, such variables, including in-group identity, behavioral norms, in-group goals, and collective efficacy, can influence pro-environmental donations independently and together. Moreover, our poster design referenced Howe but was not an exact replication [27]. In terms of manipulated group-appeal elements, this study had even fewer elements, and the poster designed by Howe et al. included a circular layout of people holding hands. Owing to the differences in the cultural contexts of the two studies, this study aimed to explore whether the inclusion of group elements could work. Future research could explore whether the number and intensity of elements could produce different effects. In any case, the results of the present study demonstrate that even with a small number group-appeal elements, working-together normative appeals still produce a greater willingness to donate, although this may also make the working-together normative appeals less dominant in a chain mediation model.

### 5.3. Theoretical and Practical Implications

This study innovatively constructed a chain mediation model, drawing from the SIMPEA, the theory of planned behavior, and the mind sponge theory to examine the effectiveness and underlying mechanisms of working-together normative appeals in fostering pro-environmental donations. From one perspective, this approach offered insight into the development of pro-environmental behaviors in donations through the lens of group dynamics, highlighting the internal mechanisms influenced by working-together normative appeals. However, the results not only corroborated the SIMPEA but also enriched and broadened the application of the theory of planned behavior in the context of pro-environmental actions.

This study underscores that working-together normative appeals are particularly effective in collectivist cultures. As a country with a collectivist culture, individuals are part of a cultural context that prioritizes group goals over individual goals, where the role and responsibilities of individuals within a group are highly valued [30]. Therefore, individuals in a collectivist culture may be more inclined to act through collective efforts, particularly in addressing global issues such as environmental protection. In the context of China’s collectivist culture, promoting pro-environmental behaviors may require considering cultural factors and incorporating a collective perspective to better motivate and harness the potential of collective action. “Working-together” normative appeals have been scarcely studied as a means of motivating individual group identity [27]. This study demonstrates the unique role of “Working-together” normative appeals in a collective cultural context. In such societies, leveraging social norms to motivate individuals toward pro-environmental choices and actions becomes more impactful when coupled with the notion of communal effort. This “working together” message encourages participation in environmental protection, fostering a harmony between human society and environmental management. Moreover, the findings lay the groundwork for developing a theory of pro-environmental behavior that aligns with the cultural nuances of collectivism. However, it is yet to be determined whether these results are consistent across different cultural contexts. This research opens avenues for future studies to explore the cross-cultural applicability of these findings.

Practically, as “nudge” has gradually become an effective strategy for promoting environmental behavior in recent years [59], this study provides a new way to publicize and advocate pro-environmental donations. From a cognitive perspective, the use of textual and pictorial messages such as “Let’s work together” and “Join us” in posters can effectively circumvent human loss aversion by utilizing the gain–loss framing effect [60], highlighting the social gain attributes of pro-environmental behaviors and promoting pro-environmental decision making, accentuating the social benefits of pro-environmental behaviors and promoting pro-environmental decision making.

In other words, this study provides a more “economical” pro-environmental behavior intervention that can increase intentions towards pro-environmental donations and promote pro-environmental donations while using fewer group appeals. Moreover, this intervention is low-cost, convenient, and accessible, which is conducive to its implementation and effectiveness as well as its widespread promotion and application, meaning that pro-environmental donations can become collective actions for the entire society. This also provides insights into how the fields of education and consumption can help in building internal societal behaviors for ecological surplus [61]. Specifically, by invoking a call for working-together in education and emphasizing a joint endeavor for a better ecological future in consumption, it is possible to maximize environmental benefits in society in a simple manner.

### 5.4. Limitations and Future Directions

There are some limitations in this study. First, working-together normative appeals utilize only one of many social norms. This study does not take into account how the complex interplay between multiple social norms influences individuals to make pro-environmental decisions. The role of different types and levels of social norms in pro-environmental donations can be explored from a broader perspective in the future. Second, the participants in this study were college students, who have higher pro-environmental intentions than other populations [62]. In fact, the environmental awareness of adolescents is also evolving, and they face challenges in translating intentions into behaviors [63]. The robustness of the paradigm could be tested by examining whether the working-together normative appeals can enhance the intentions and behaviors of pro-environmental donations among a wider group of people, such as adolescents. Additionally, there were differences in tone between the posters of the working-together appeals group and the control group in addition to whether or not they included a call to work together, which may have led to some bias. Future research should explore the differences brought about by several different messages to obtain more robust results. Although donations to environmental organizations can be somewhat indicative of a person’s pro-environmental level, this study did not investigate whether working-together normative appeals could play an important role in daily pro-environmental behaviors. In the future, it could be explored whether this kind of norm for pro-environmental behaviors could trigger changes in daily life [64]. Finally, the use of donations to measure pro-environmental behaviors assumes a certain level of material wealth and socioeconomic status, among other factors [65,66,67]. In the future, longitudinal tracking or experimental designs could be used to explore how the interactions of individual values and socioeconomic status with social norms affect pro-environmental behaviors.

## 6. Conclusions

Working-together normative appeals significantly enhance the willingness of actual behaviors to donate to environmental initiatives. Perceived behavioral control and intentions towards pro-environmental donations act as mediators between the categorization of social norms and pro-environmental donations, with intentions towards pro-environmental donations playing a notably significant role. Consequently, working-together normative appeals are demonstrated to boost intentions towards pro-environmental donations via perceived behavioral control, and directly augment pro-environmental donations by elevating intentions towards pro-environmental donations. This dual effect underscores the efficacy of working-together appeals in promoting environmentally responsible actions.

## Figures and Tables

**Table 1:** Path analysis of mediation model.

	Effect Value	SE	95% LLCI	95% ULCI
Total effect	0.33	0.08	0.16	0.49
Direct effect	0.16	0.08	−0.01	0.34
Total mediating Effect	0.17	0.05	0.08	0.28
Path1	0.03	0.02	−0.01	0.08
Path2	0.02	0.01	0.01	0.05
Path3	0.12	0.04	0.05	0.22

## Data Availability

The data that support the findings of this study are openly available in [Figshare] at URL http://doi.org/10.6084/m9.figshare.25347487 (accessed on 22 March 2024).
